# Flow-Xl: a new facility for the analysis of crystallization in flow systems

**DOI:** 10.1107/S1600576724006113

**Published:** 2024-08-19

**Authors:** T. D. Turner, C. O’Shaughnessy, X. He, M. A. Levenstein, L. Hunter, J. Wojciechowski, H. Bristowe, R. Stone, C. C. Wilson, A. Florence, K. Robertson, N. Kapur, F. C. Meldrum

**Affiliations:** a School of Chemistry University of Leeds LeedsLS2 9JT United Kingdom; b Université Paris-Saclay, CEA, CNRS, NIMBE, LIONS 91191Gif-sur-Yvette France; c Rigaku Europe SE, Hugenottenallee 167, 63263Neu-Isenburg, Germany; d Department of Chemistry University of Bath Bath United Kingdom; e Centre for Continuous Crystallisation University of Strathclyde Glasgow United Kingdom; f Faculty of Engineering, University Park University of Nottingham NottinghamNG7 2RD United Kingdom; g School of Mechanical Engineering University of Leeds LeedsLS2 9JT United Kingdom; HPSTAR and Harbin Institute of Technology, People’s Republic of China

**Keywords:** powder X-ray diffraction, Raman spectroscopy, *in situ* characterization, polymorphic transformation, crystal nucleation, crystal growth, flow systems

## Abstract

Characterization of crystallization processes *in situ* is of great importance to furthering knowledge of how nucleation and growth processes direct the assembly of organic and inorganic materials in solution and, critically, understanding the influence that these processes have on the final physico-chemical properties of the resulting solid form. With careful specification and design, as demonstrated here, it is now possible to bring combined X-ray diffraction and Raman spectroscopy, coupled to a range of fully integrated segmented and continuous flow platforms, to the laboratory environment for *in situ* data acquisition for timescales of the order of seconds. The facility used here (Flow-Xl) houses a diffractometer with a micro-focus Cu *K*α rotating anode X-ray source and a 2D hybrid photon-counting detector, together with a Raman spectrometer with 532 and 785 nm lasers. An overview of the diffractometer and spectrometer setup is given, and current sample environments for flow crystallization are described. Commissioning experiments highlight the sensitivity of the two instruments for time-resolved *in situ* data collection of samples in flow. Finally, an example case study to monitor the batch crystallization of sodium sulfate from aqueous solution, by tracking both the solute and solution phase species as a function of time, highlights the applicability of such measurements in determining the kinetics associated with crystallization processes. This work illustrates that the Flow-Xl facility provides high-resolution time-resolved *in situ* structural phase information through diffraction data together with molecular-scale solution data through spectroscopy, which allows crystallization mechanisms and their associated kinetics to be analysed in a laboratory setting.

## Abstract 2

A new *in situ* facility (Flow-Xl) for the characterization of crystallization systems in flow using combined *in situ*X-ray diffraction and Raman spectroscopy is presented. This article highlights commissioning and calibration experiments on case-study systems that stress the utility of the instrument for extracting reproducible qualitative and quantitative phase information during crystallization processes.

## 1. Introduction

Crystallization is fundamental to industrial science and engineering over an extensive range of disciplines including pharmaceuticals, nanoparticle synthesis, agrochemical production and ceramics manufacturing, as well as a myriad of processes in the environment and in biology. Characterization of these complex phenomena is required in order to understand and ultimately control these crystallization processes. However, for the widespread case of crystallization from solution, *ex situ* characterization presents multiple sampling problems, where isolation and drying of the product can induce phase changes and aggregation. *In situ* methodologies are therefore required to adequately characterize the nucleation and growth processes that are associated with crystallization pathways.

The availability of modern synchrotron radiation (SR) facilities has been instrumental in the study of crystallization phenomena, where the high photon flux, tuneable radiation energy and fast counting area detectors that these facilities offer have facilitated a range of diffraction, scattering and spectroscopic studies. This has allowed researchers to probe structural and chemical changes at much superior temporal resolutions than can be achieved with more traditional laboratory instruments, which enables measurement of rapid kinetic processes associated with nucleation and growth processes during crystallization (Levenstein, Kim *et al.*, 2020 ▸; Durelle *et al.*, 2022 ▸; Polte, Erler, Thünemann, Emmerling & Kraehnert, 2010 ▸). These methods have been applied to a range of systems, where they have, for example, revealed the kinetics of nanoparticle and metal–organic framework synthesis and growth (Chambers *et al.*, 2022 ▸; He *et al.*, 2021 ▸), the solution-mediated phase transformations of amorphous and crystalline organic phases (Nguyen *et al.*, 2017 ▸; Gnutzmann *et al.*, 2014 ▸; Levenstein, Wayment *et al.*, 2020 ▸), and fundamental structural parameters associated with nucleation processes (Jawor-Baczynska *et al.*, 2013 ▸). SR small-angle/wide-angle X-ray scattering techniques have been particularly valuable in probing the early stages of crystallization (Davey *et al.*, 2002 ▸; Toroz *et al.*, 2015 ▸; Whittaker *et al.*, 2017 ▸). There have also been significant advances in the use of serial crystallography for high-resolution and high-throughput crystallographic analysis of macromolecules (Pinker *et al.*, 2013 ▸; Calvey *et al.*, 2019 ▸; Beyerlein *et al.*, 2017 ▸), which has been achieved by coupling novel microfluidic devices first to X-ray free-electron lasers (Sellberg *et al.*, 2014 ▸) and then to SR end stations (Stellato *et al.*, 2014 ▸).

However, the use of SR for analysis of crystallization pathways is not without its limitations. There are inevitably long waiting times to access facilities, and restrictions on beam time often limit the complexity and duration of experiments. Access to experimental equipment through the hutch is either time consuming and inconvenient or necessitates complex remote operation/automation. The possibility of conducting measurements in a laboratory setting is therefore extremely attractive, and the continued improvements in the field of laboratory diffraction instrumentation over the past two decades are now making this dream a reality (Polte, Erler, Thünemann, Sokolov *et al.*, 2010 ▸; Levenstein *et al.*, 2022 ▸; Chen *et al.*, 2015 ▸; Radajewski *et al.*, 2023 ▸). Modern laboratory diffractometers have much improved photon flux at the sample thanks to advances in X-ray source technologies, including highly efficient micro-focus rotating anodes and liquid metal jet anode materials (Skarzynski, 2013 ▸); these make energies of 8–25 keV routinely accessible. Parallel improvements in detector technology and computing power have also resulted in laboratory diffractometers using 2D hybrid photon counting (HPC) detectors as standard practice, yielding greatly improved noise reduction and counting statistics.

Notably, these recent technological improvements have facilitated laboratory-based *in situ* studies of materials characterization that would traditionally have been performed at an SR facility, such as in the fields of electrochemistry and energy research, where powder diffraction has revealed the crystallographic variations inherent within battery materials during voltage cycling (Geßwein *et al.*, 2022 ▸). *In situ* diffraction, combined with Raman spectroscopy, has provided detailed information on the chemical and structural fluctuations within catalyst materials during reactions at elevated pressures (Cats & Weckhuysen, 2016 ▸). The use of *in situ* laboratory-based diffraction has also been applied to study the preferential enrichment mechanism of chiral organic compounds through measurement of a solid–solid phase transition during solution crystallization (Takahashi *et al.*, 2017 ▸). Parallel to these advances in measurement systems (Levenstein *et al.*, 2022 ▸), significant progress in the fields of micro- and milli-fluidics for continuous crystallization devices has provided very well controlled and reproducible environments (mixing, supersaturation, removal of surface interactions *etc*.) for probing the nucleation and growth mechanisms associated with solution crystallization (Robertson *et al.*, 2016 ▸). This has been successfully applied to study the solid-form landscape of small organic molecules by identifying the early stages of crystal formation and growth of the metastable phase of succinic acid through *in situ* Raman spectroscopy (Pallipurath *et al.*, 2020 ▸).

This article describes the design, construction and commissioning of a new laboratory facility – Flow-Xl – that enables the time-resolved characterization of crystallization processes in highly controlled solution environments under continuous flow and data-acquisition times of the order of seconds. This is achieved by coupling state-of-the-art laboratory X-ray diffraction (XRD) and Raman spectroscopy instruments to a range of fully integrated flow platforms. The flow crystallization platforms developed will allow users to create well defined experiments in continuous or segmented flow, which represent common crystallization environments in nature and industrial processing. Commissioning experiments highlight the sensitivity of the two instruments for time-resolved *in situ* data collection of samples under flow and also show that the limit of detection (LOD) is such that small changes in solid/solution concentration can be captured, which is vital in enabling characterization of crystallization mechanisms and solid phase transformations with good time resolution. Finally, an example case study to monitor the batch crystallization of Na_2_SO_4_ from aqueous solution, by tracking both the solute and solution phase species as a function of time, highlights the applicability of such measurements in determining the kinetics associated with crystallization processes.

## 2. Materials and methods

### 2.1. Materials

The following materials were used: silver behenate (Alfa Aesar), sodium sulfate, potassium nitrate, l-glutamic acid BioUltra ≥ 99.5% (Sigma), paracetamol = 98.0–102.0% (Sigma), calcium carbonate ACS reagent ≥ 99.0% (Sigma), theophylline anhydrous ≥ 99% (Sigma), deionized water, iso­propyl alcohol reagent grade ≥ 99.5% (Fisher). Reagents were used as received, without further purification.

### 2.2. XRD calibration of suspensions of solids

Saturated solutions of the relevant material [calcium carbonate (calcite, >50 µm particle size) and theophylline (form II >100 µm particle size)] were prepared at room temperature in deionized water and iso­propyl alcohol, and transferred to a jacketed glass reactor vessel (Duran) by passing the solution though a 0.22 µm filter. A known mass of crystals was weighed using a four-figure balance and then transferred to the saturated solution to form a standard of known slurry density (wt%). The slurry was agitated using a 40 mm magnetic stirrer and pumped to a measurement cell through a flow loop of fluorinated ethylene propylene (FEP) tubing of 3/16′′ outer diameter (OD), using a four-roller peristaltic pump set to 150 rev min^−1^. Diffraction data were collected in transmission geometry through a 2 mm OD borosilicate glass capillary tube with a wall thickness of 10 µm for 10 s, with a sample-to-detector distance of 80 mm and divergence slit optics set to 10 mrad.

### 2.3. Raman calibration of solution concentration

Standard solutions of known concentrations of KNO_3_ and Na_2_SO_4_ in deionized water and paracetamol in dry ethanol were prepared, where the respective solute solids were weighed into clean vials using a four-figure balance. The relevant solvent was then weighed into the vials to make up the required concentration using a micropipette, before being transferred to a 1 mm quartz glass cuvette cell for Raman analysis.

### 2.4. Raman calibration of suspensions of solids

Saturated solutions of the relevant solute (calcite of >50 µm particle size and l-glutamic acid of >100 µm particle size) were prepared at room temperature and transferred to a jacketed glass reactor vessel (Duran) by passing the solution though a 0.22 µm filter. A known mass of crystals of the solute were weighed using a four-figure balance and then transferred to the saturated solution to form a standard of known slurry density (wt%). The slurry was agitated using a 40 mm magnetic stirrer and pumped to a measurement cell through a flow loop of FEP tubing of 3/16′′ OD, using a four-roller peristaltic pump set to 150 rev min^−1^ at an approximate flow rate of 220 ml min^−1^.

### 2.5. Collection of Raman data

Raman data were collected with a Labram HR Evolution microscope using a 10× objective, where data-acquisition times were 10 s, and averaged over three acquisitions for the spectral range 300–1200 cm^−1^. A 532 nm laser was used during data collection at 100% power setting with a 1800 (450–850 nm) grating size.

## 3. Results and discussion

### 3.1. Facility overview

**Figure 1:**
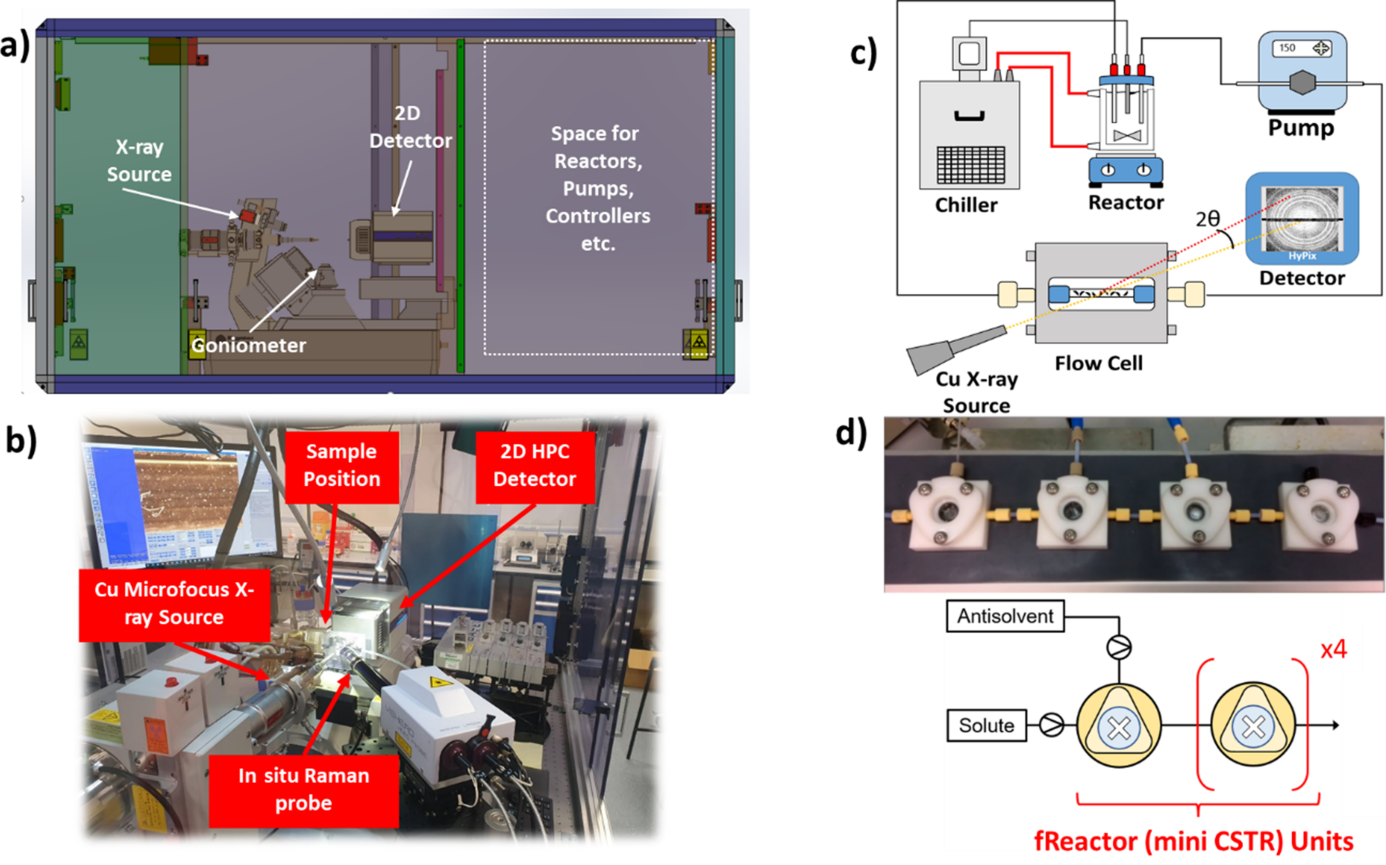
(*a*) A mechanical drawing of the XtaLAB Synergy Custom X-ray diffractometer, highlighting the large enclosure that houses the Cu rotating anode X-ray source, goniometer and 2D HPC detector. (*b*) A photograph of the inside of the X-ray enclosure highlighting the main components and the Raman probe. (*c*) A typical batch crystallization flow loop with capillary flow cell setup as shown in (*b*). (*d*) The continuous crystallization reactor geometry, including fReactor design (Guan *et al.*, 2020 ▸; Manson *et al.*, 2019 ▸; Chapman *et al.*, 2017 ▸).

The Flow-Xl laboratory has a state-of-the-art Rigaku XtaLAB Synergy Custom X-ray diffractometer, with a large radiation enclosure built to house a range of complex flow-system setups (Fig. 1 ▸). The enclosure measures 1.86 × 1.42 m and contains an optical breadboard with M6 (25 × 25 mm standard) tapped holes for fixing equipment in position and/or building stages for mounting pumps, controllers, reactors and other ancillary components. The diffractometer houses an MM007-HF (Cu, 1.54 Å) micro-focus rotating anode X-ray source, equipped with VariMax Very High Flux X-ray optics that yield a beam of 150 µm diameter [full width at half-maximum (FWHM)] and 10 mrad divergence with ultra-high brightness at the sample, >2.0 × 10^11^ photons s^−1^mm^−2^, optimized for data collection from the weakest of diffracting samples. The system is also configured with an HPC X-ray detector, which has a pixel size of 100 × 100 µm, a high dynamic range, fast readout speed (up to 100 Hz in shutter-less collection mode) and extremely low noise. It is set up to collect low-noise data quickly, which is ideal for *in situ* diffraction studies of samples in flow (Le Magueres *et al.*, 2019 ▸). For the purpose of sample translation, the diffractometer has a motorized stage accessory, the XtalCheck-S, which enables sample movement of up to 10 mm s^−1^ in the *x*, *y* and *z* directions and rotation about Ω. This allows the sample to be centred on the goniometer and monitored via a high-resolution colour video camera. The XtalCheck-S accepts any sample within the dimensions of a standard Society of Biomolecular Screening (SBS) crystallization plate (128 × 86 × 15 mm), and 3D-printed sample holders can also be manufactured to mount such samples.

Additionally, the facility contains a HORIBA Jobin Yvon Labram HR Evolution Raman Spectrometer that is equipped with green (532 nm) and red (785 nm) lasers with respective ultra-low frequency modules to allow measurements in the sub-100 cm^−1^ region. Two SuperHead fibre optic probes facilitate *in situ* measurements in tandem with the X-ray diffractometer, where the probe is mounted onto an *x*, *y*, *z* stage for sample-focusing purposes.

### 3.2. Facility sample environments

**Figure 2:**
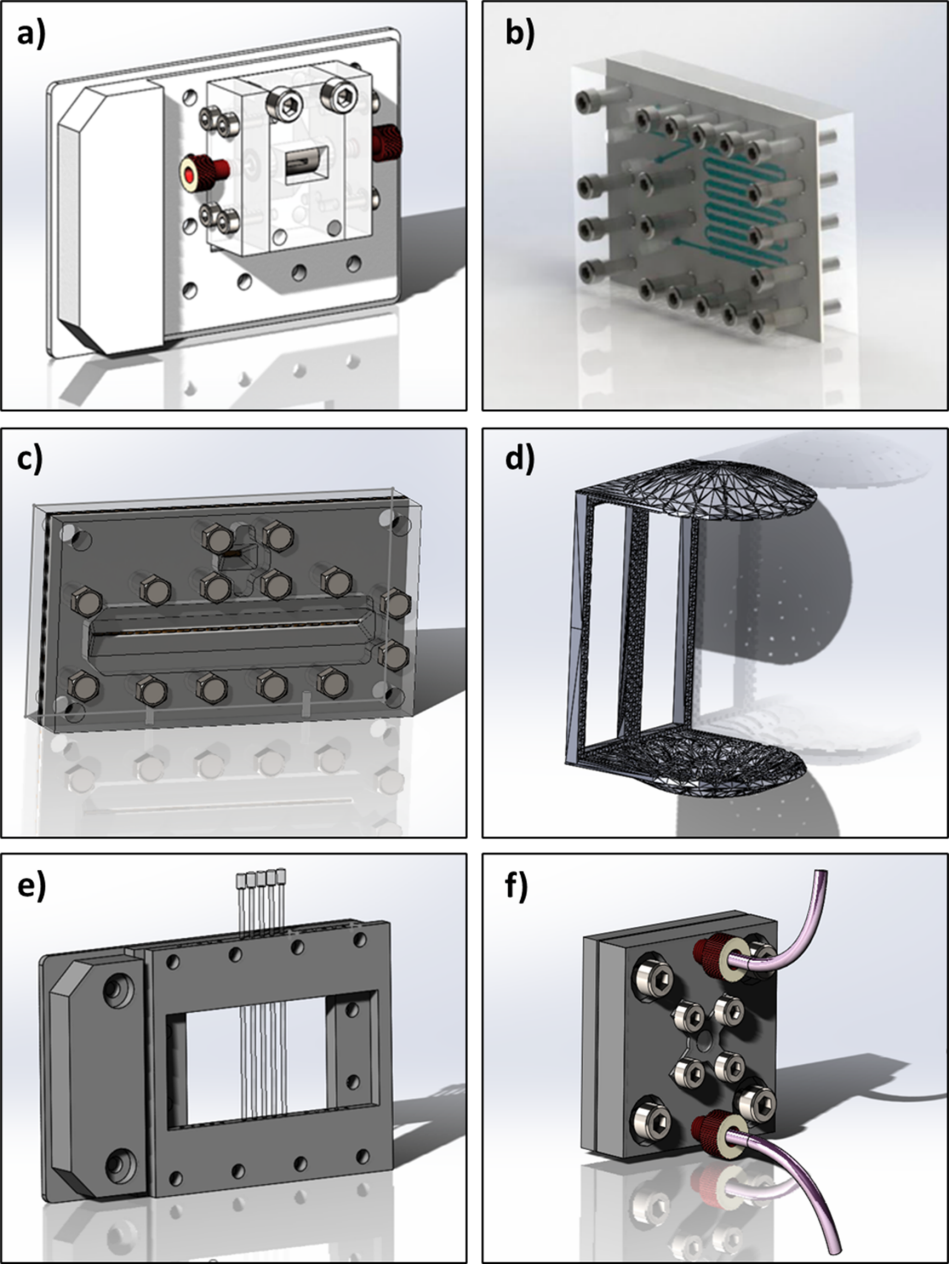
(*a*) A slurry flow cell with a 1.5/2.0 mm borosilicate glass capillary tube for use with process reactors/continuous crystallization setups (Turner *et al.*, 2018 ▸). (*b*) An oscillatory baffled continuous crystallization chip for microfluidic experiments (González Niño *et al.*, 2019 ▸; reproduced courtesy of Taylor & Francis Ltd, https://www.tandfonline.com). (*c*) A microfluidic droplet device for continuous flow crystallization under controlled conditions (Levenstein *et al.*, 2019 ▸). (*d*) An acoustic levitator (Marzo *et al.*, 2017 ▸) used to generate single liquid droplets for evaporative crystallization in controlled environments. (*e*) A 3D-printed capillary-tube holder for 0.5–2.0 mm capillary tubes used for powder characterization of standard materials. (*f*) A variable humidity cell for monitoring changes to solid powders as a function of relative humidity.

The Flow-Xl facility has several sample environments available to users (Fig. 2 ▸). The focus is on liquid samples, but some solid sample holders are also available. Fig. 2 ▸(*a*) shows a flow device for use with process reactors or continuous crystallization setups. This provides a sampling point to measure diffraction or spectroscopy data of a flowing solution or suspension of particles. The device, which is machined from stainless steel, comprises a main cell body that houses a cylindrical capillary tube holder and two side sections that allow the device to be connected to a solution flow loop through a pumping mechanism, *e.g.* a peristaltic pump or syringe drivers. The capillary cartridge system allows any size capillary tube, and hence sampling path length, to be realized and additionally facilitates rapid changeover if breakages/blockages occur during measurements. Further details of this cell are provided elsewhere (Turner *et al.*, 2018 ▸).

Fig. 2 ▸(*b*) shows an example of a continuous oscillatory baffled chip reactor for improved solution stream mixing in continuous flow reactive/anti-solvent crystallization experiments, as described previously (González Niño *et al.*, 2019 ▸). Additionally, a number of segmented flow devices are available that allow crystallization in well controlled droplet environments to be studied, such as the microfluidic chip device shown in Fig. 2 ▸(*c*). The device consists of a laser-cut poly(tetra­fluoro­ethyl­ene) serpentine chip to provide the reactor geometry, which is then sandwiched between polyimide windows and silicon seals to provide a measurement window for X-rays (Levenstein *et al.*, 2019 ▸). Fig. 2 ▸(*f*) shows an acoustic levitator used for levitating 2–10 µL solution droplets, which provides a well controlled geometry for studying crystallization during evaporation of the droplets in air (Marzo *et al.*, 2017 ▸).

A capillary tube holder [Fig. 2 ▸(*e*)] was designed and 3D printed from polycarbonate with 2 mm diameter holes in the main body for glass capillary tubes to be inserted. This holder was adapted from the dimensions of a standard SBS well plate to fit the XtalCheck-S system of the diffractometer. A solid sample cell for variable humidity experiments [Fig. 2 ▸(*f*)] was also designed and machined from stainless steel. The latter comprises two 50 mm^2^ plates that fit together to contain a steel ring and hold a perforated polyimide tube (0.5–2 mm) in place for sample containment. The whole device is sealed using two O-rings seated on both sides of the sample holder with two Kapton windows at the entrance and exit of the device, again sealed with O-rings (see Section S1 of the supporting information for further details). The humidity inside it can be controlled using a humidity generator and controller, which can be connected to the front of the cell through two ¼–28 threaded holes. This device can also be mounted to an SBS plate adapter through M6 mounting holes to fit onto the XtalCheck-S system of the diffractometer.

### 3.3. Calibration experiments

#### 3.3.1. Diffractometer calibration and commissioning

**Figure 3:**
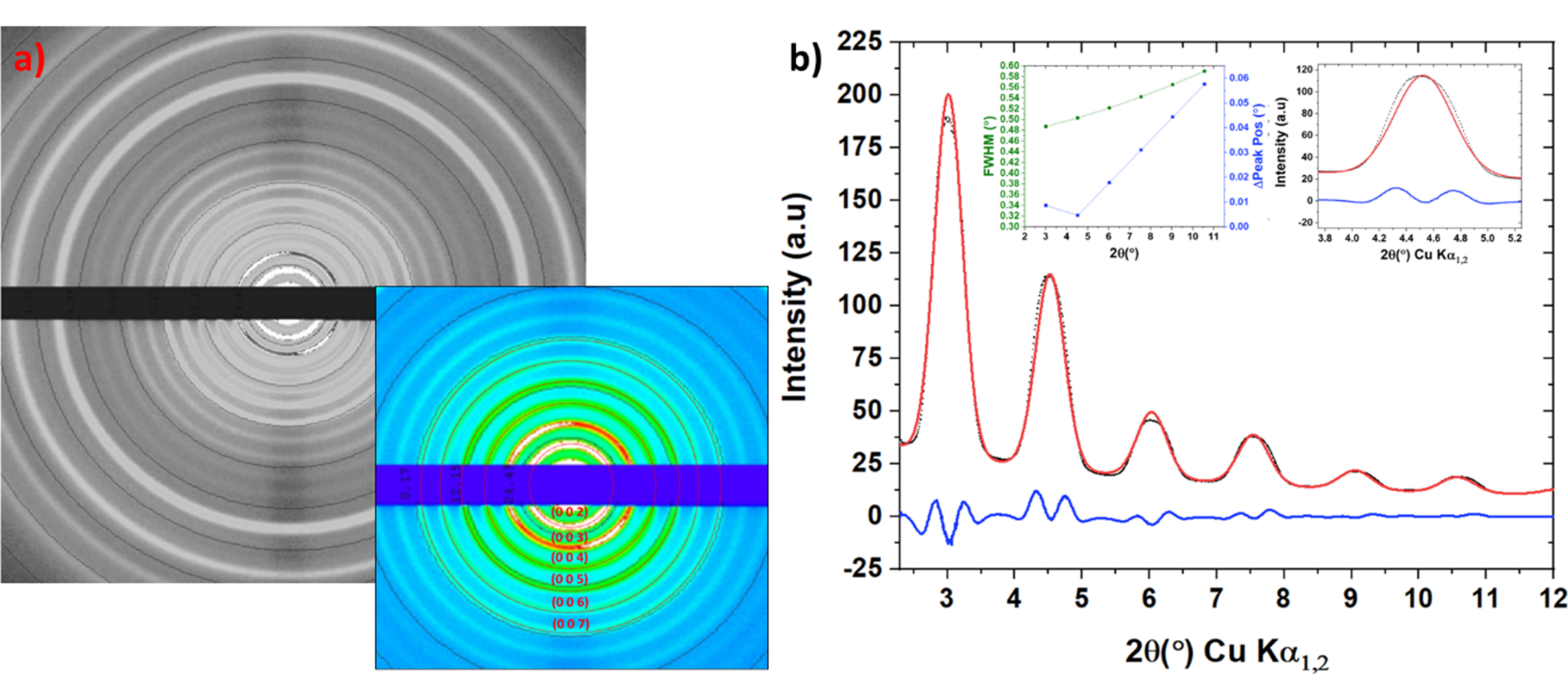
(*a*) Two-dimensional diffraction patterns of silver behenate powder collected in a 1 mm borosilicate capillary tube at 10 mrad optics. The (00*l*) planes are highlighted for clarity in the inset. (*b*) Rietveld fitting of the silver behenate diffraction pattern; the experimental data are in black, the fitted profile is in red and the difference plot is in blue. Inset is the 004 diffraction peak to highlight peak shape and the FWHM and Δ peak position against 2θ.

The HPC detector of the diffractometer was calibrated using silver behenate, which was ground to a powder using a pestle and mortar and mounted into a 1 mm diameter, 10 µm wall thickness, borosilicate glass capillary tube. The sample was placed in a 3D-printed capillary holder [Fig. 2 ▸(*a*)] and mounted on the XtalCheck-S accessory system on the goniometer. The collected 2D diffraction image is shown in Fig. 3 ▸(*a*) and highlights the well defined diffraction rings of the (00l) planes. Following data reduction and integration, a profile-fitting routine was performed to obtain fitted peak positions, which were compared with the corresponding calculated peak positions obtained via the known crystal structure of the material (Huang *et al.*, 1993 ▸; Blanton *et al.*, 2011 ▸). The analysis of these data is summarized in Table 1 ▸ and shows that the mean positional difference in peak 2θ values is 0.0273° between the measured data and the calculated values. This provides a good estimate of the error in absolute measured 2θ values of the instrumen. The peak profile shapes were very symmetrical and Gaussian-like, with little evidence of peak asymmetry. The angular dependence of the FWHM values [Fig. 3 ▸(*b*) inset] increased over the measured 2θ range (∼3–11° 2θ) from 0.48 to 0.58°; additionally, the calculated Δ peak position, the difference between the crystal structure and the fitted peak positions, was found to be between 0.01 and 0.06°. This provides confidence in the instrument indexing ability for complex mixtures of polymorphic phases where peak overlap is often problematic, particularly for organic crystal systems.

**Figure 4:**
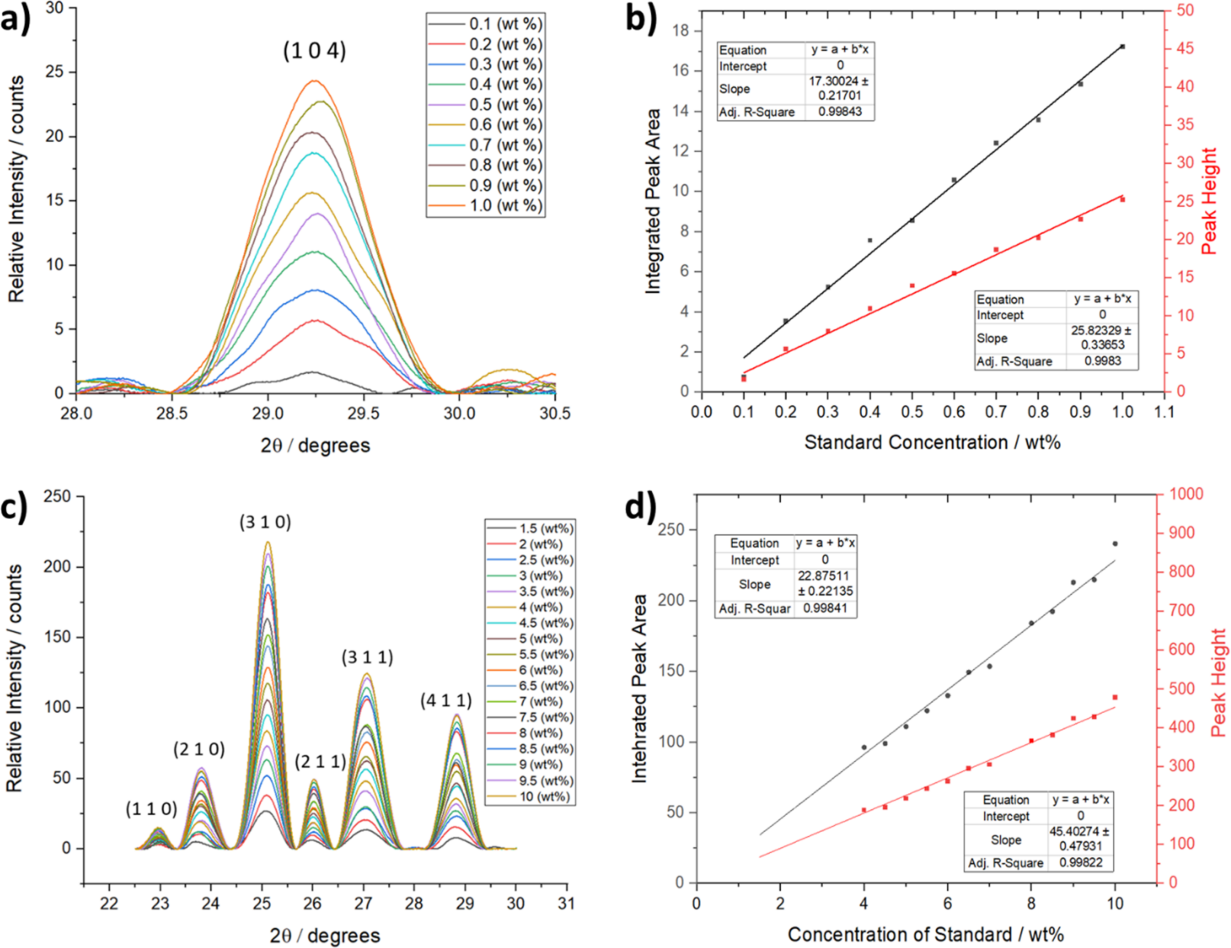
Diffraction calibration standards showing (*a*) the concentration dependence of the 104 diffraction peak of calcite for slurry concentrations of 0.1–1.0 wt% under flow using the slurry cell shown in Fig. 2 ▸(*c*) with a 2 mm path length, (*b*) linear calibration curves of the integrated calcite 104 peak areas and heights as a function of concentration (intercepts were set to 0 and the calculated standard error of the slope is also provided), (*c*) the concentration dependence of the diffraction pattern of theophylline form II at slurry concentrations of 1.5–10.0 wt% in iso­propanol/water solutions, and (*d*) linear calibration curves of the integrated theophylline form II 310 diffraction peak areas and heights as a function of concentration, highlighting the fitted linear equation parameters.

Fig. 4 ▸ highlights the XRD calibration data for two contrasting model systems, CaCO_3_ (calcite) and theophylline form II, which were chosen as representatives of the crystalline materials commonly studied using the Flow-Xl system. Fig. 4 ▸(*a*) shows the concentration-dependent diffraction data collected using the 2 mm borosilicate glass capillary flow cell [Fig. 2 ▸(*a*)] for 0.1 to 1.0 wt% calcite aqueous slurries. The LOD is ∼0.1 wt% for 10 s data-collection times under these conditions. Fig. 4 ▸(*b*) shows a good linear correlation between the concentration and the 104 peak heights and integrated areas following the application of a peak-fitting algorithm.

Fig. 4 ▸(*c*) shows the concentration-dependent diffraction data for 1.5 to 10.0 wt% slurries of theophylline form II in iso­propyl alcohol collected under the same conditions as for calcite. The intensity of many of the diffraction peaks increases with concentration, and a linear correlation of concentration with peak height and area was found following peak fitting and integration of the 301 diffraction peak. The LOD for the theophylline form II samples was 0.6 wt% under the conditions studied (see Section S2). Overall, the data show that the XRD instrument can provide quantitative solid phase information down to low particle suspension concentrations under flow conditions. Additionally, these experiments have demonstrated the measurement sensitivity even at short acquisition times of 10 s, which would allow phase-transformation information to be obtained in real time, at least for samples within this particle size range of >50 µm.

#### 3.3.2. Raman spectrometer calibration and commissioning

**Figure 5:**
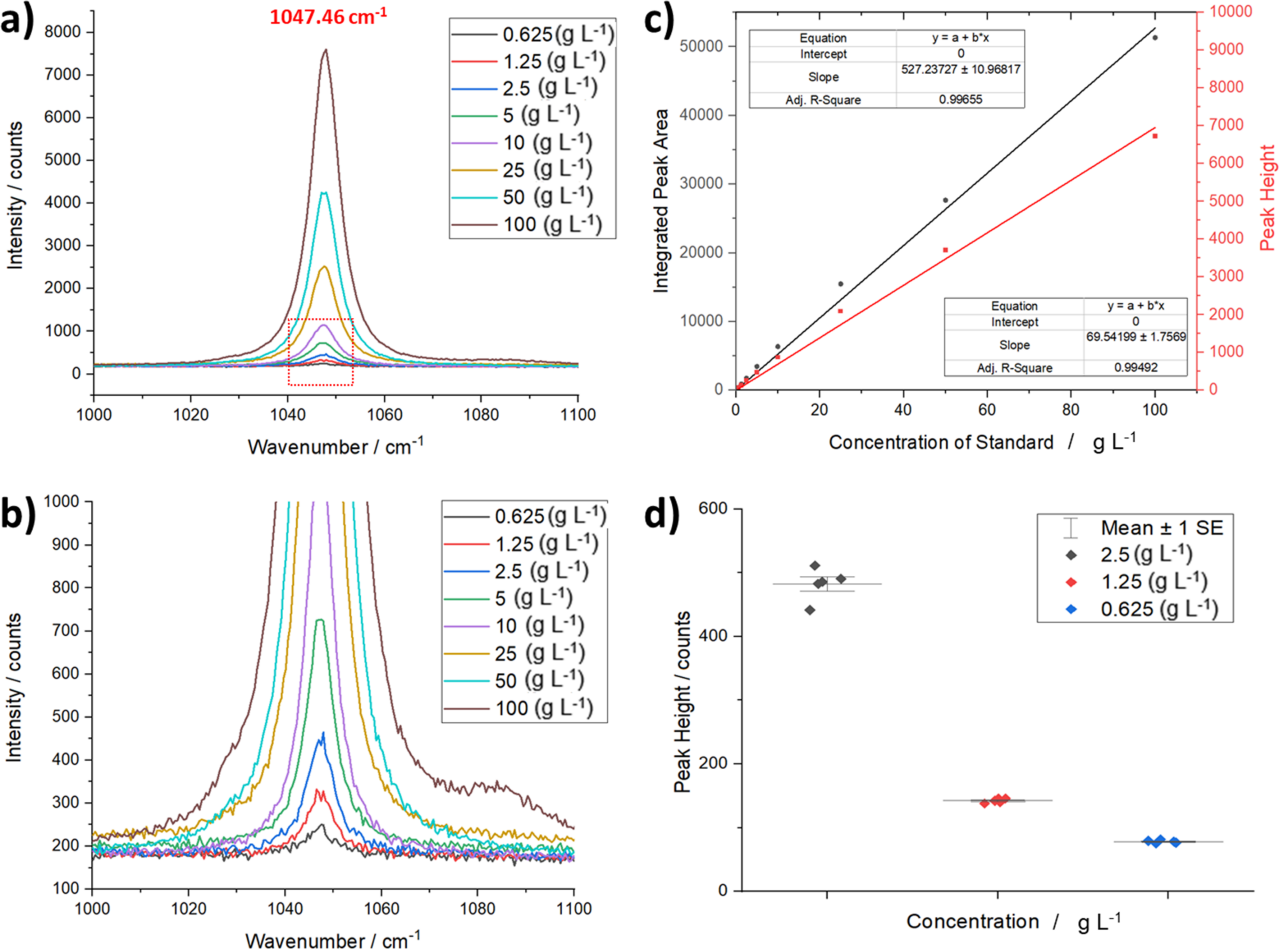
(*a*) Raman calibration standards for KNO_3_ aqueous solutions highlighting the concentration dependence of the 1047.46 cm^−1^ peak in the spectra. (*b*) Magnification of the dotted box in (*a*) showing the LOD for KNO_3_ solutions of ∼0.625 g L^−1^. (*c*) Linear calibration of the peak height and area versus solution concentration of the 1047.46 cm^−1^ peak. (*d*) Interval plots of the peak heights for the 0.625, 1.25 and 2.5 g L^−1^ KNO_3_ solutions, showing the spread of data for five replicate measurements used to calculate the standard deviation and RSD.

The Raman spectrometer provides complementary chemical and structural information to the diffractometer, and hence provides a useful tool for probing not only the solid phase composition but also the molecular details of the solution and solvated species. This approach is particularly useful when performing both qualitative and quantitative analyses of processes such as nucleation, growth and structural phase transitions. Therefore, several commissioning experiments were performed to explore the sensitivity of the Raman instrument in identifying phases and, critically, quantifying them in flow. Fig. 5 ▸ highlights Raman data collected for a model compound, KNO_3_, in aqueous solutions. The concentration range 0.625–100 g L^−1^ was studied using the slurry flow cell (Fig. 2 ▸) with 10 s data-collection times in transmission geometry. Fig. 5 ▸(*a*) shows the concentration dependence of the 1047.46 cm^−1^ Raman band, which relates to the solvated species of KNO_3_, and Fig. 5 ▸(*b*) expands the lower concentration for clarity and shows that the LOD for the KNO_3_ solution state species is ∼0.625 g L^−1^ under these measurement conditions. Fig. 5 ▸(*c*) shows the linear calibration curve of the integrated peak areas and heights following baseline correction and integration of the 1047.46 cm^−1^ band using a Gaussian function, and reveals good fitting of a linear function with an *R*^2^ value of 0.997.

The reproducibility of these measurements is an important consideration, particularly when expanding this type of analysis to the kinetics of crystallization processes. A statistical error analysis was therefore applied to repeat measurements for a range of concentrations. Fig. 5 ▸(*d*) highlights the spread of data for the peak heights of the 1047.46 cm^−1^ KNO_3_ solution peak at concentrations of 0.625, 1.25 and 2.5 g L^−1^ for five replications of the same data-collection strategy. The interval plots show that the spread in peak heights is fairly small around the mean values for all concentrations. The mean calculated relative standard deviation (RSD) is 2.92% for the peak heights and 3.69% for the integrated peak areas over the range 0.625–100 g L^−1^.

**Figure 6:**
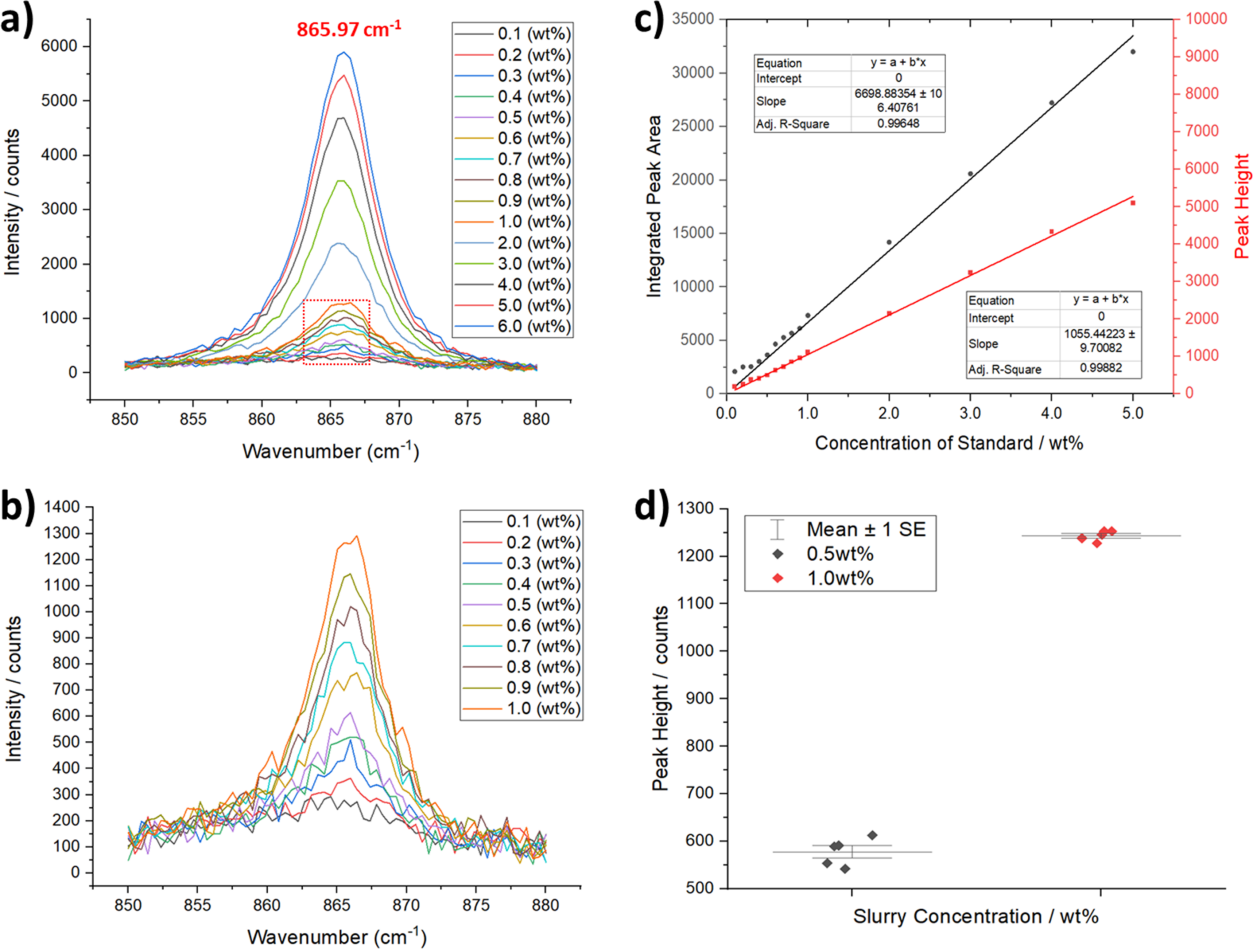
(*a*) Raman calibration standards for β-l-glutamic acid aqueous slurries showing the concentration dependence of the 865.97 cm^−1^ peak of the solid-state spectra with increasing slurry mass. (*b*) Magnification of the dotted box in (*a*) showing that the LOD for the β phase of l-glutamic acid slurries is ∼0.1 wt%. (*c*) Linear calibration curve for the peak height and integrated peak area of the 865.97 cm^−1^ peak. (*d*) Interval plots of the peak heights for the 0.5 and 1.0 wt% l-glutamic acid slurries, showing the spread of data for five replicates used to calculate the standard deviation and RSD.

To assess the sensitivity of the Raman instrument in detecting and quantifying solid suspensions, a number of l-glutamic acid β form crystal slurries were prepared and circulated through the capillary flow cell [Fig. 2 ▸(*c*)]. They were analysed as for the KNO_3_ solutions, with each spectrum collected for 10 s. The concentration-dependent Raman spectra measured for the l-glutamic acid β form slurries over the concentration range 0.1–6.0 wt% are shown in Fig. 6 ▸(*a*), which highlights the 865.97 cm^−1^ Raman band associated with the stable β polymorph of l-glutamic acid. The LOD following baseline correction was found to be 0.1 wt% [Fig. 6 ▸(*b*)]. Following data reduction and integration of the 865.97 cm^−1^ Raman peak using a Gaussian function, the peak heights and integrated area were plotted against concentration. This yielded a straight line, to which linear correlations were fitted [Fig. 6 ▸(*c*)], and the *R*^2^ values were 0.998 and 0.996 for the fitting to peak heights and areas, respectively. The interval plot in Fig. 6 ▸(*d*) shows the statistical analysis for five repeat measurements of slurry samples under identical flow regimes at concentrations of 0.5 and 1.0 wt%. In common with the solution-state Raman data, the data points are narrowly distributed around the mean values of the peak height. This is further emphasized in Table 2 ▸, which shows the overall statistical analysis for the repeat measurements of the slurry data; the calculated RSDs for the peak heights and areas were 3.30 and 2.03%, respectively, over the slurry concentration range between 0.5 and 4.0 wt%.

Overall, the commissioning experiments for the Raman system show that the instrument provides a LOD for concentrations of 0.625–2.5 g L^−1^ for solution samples and 0.02–0.1 wt% for crystal suspensions under the flow regimes for the crystallization systems studied. Additionally, further crystallization systems were studied using the Raman instrument including calcium carbonate (calcite), sodium sulfate (anhydrous) and paracetamol (form I), which provided a good range of both organic and inorganic case-study systems (further details are provided in Sections S3 and S4, including LOD information for other solutes/slurries). Together with the good linear calibration curves for the concentration dependency of the solution and suspended crystal species, and the high reproducibility of these measurements, this provides confidence for future studies aimed at identifying and quantifying solution and solid species during crystallization processes under flow conditions.

### 3.4. Case study: batch crystallization of Na_2_SO_4_

The utility of the Flow-Xl system is now illustrated in a case study. The crystallization of sodium sulfate in aqueous solution was studied within a temperature-controlled batch crystallizer to represent conditions commonly found within industrial processing and manufacturing. A 250 ml solution, saturated at 30°C, was subjected to a heating/cooling cycle of 10–40–10°C at a rate of 0.2°C min^−1^ with constant stirring at 300 r min^−1^. The solution/suspension was circulated to a 2 mm borosilicate capillary flow cell through FEP tubing [Fig. 2 ▸(*c*)] using a peristaltic pump at 150 rev min^−1^ during this temperature cycling. The temperature of the tubing was not controlled and the discrepancy between the reactor temperature and the return slurry line was observed to be ∼0.2°C. Raman (external probe) and diffraction data were simultaneously collected, every 17 s for Raman data and every 18 s for XRD data, and the optics used to collect the diffraction data were set to 10 mrad to optimize the sampling volume and hence increase the diffracted signal on the detector.

**Figure 7:**
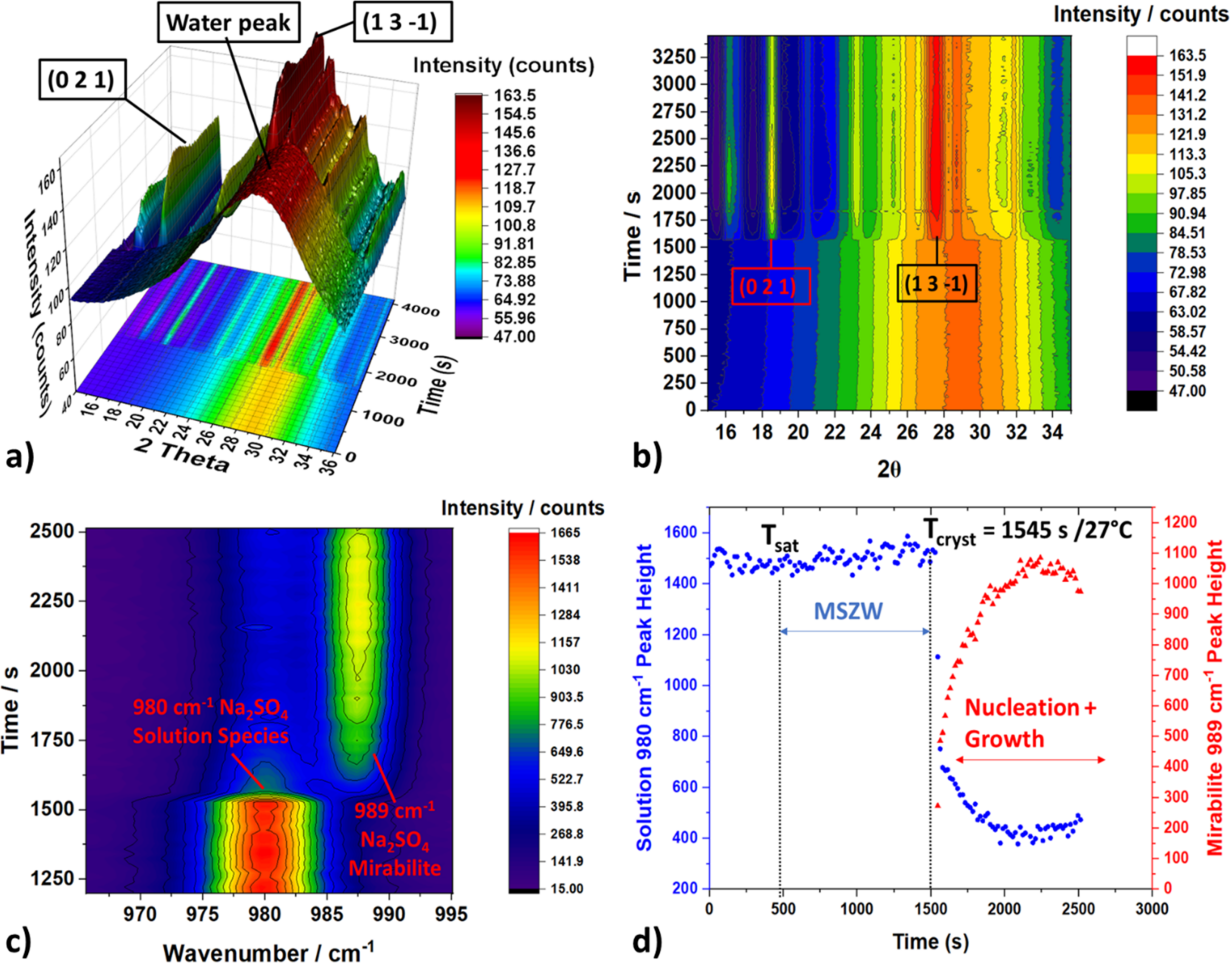
(*a*) Time-dependent diffraction data (2θ versus *t*) collected from the 2 mm borosilicate capillary flow cell during the crystallization of an aqueous 30 g/100 g Na_2_SO_4_ solution under heating–cooling cycles, highlighting the water peak corresponding to the solution phase followed by the rapid appearance of the 021 and 131 diffraction peaks after crystallization. (*b*) The same data as (*a*) in a contour-plot format for clarity. (*c*) Time-dependent Raman spectra collected during the same crystallization experiment centred on the 980 cm^−1^ peak corresponding to the solvated Na_2_SO_4_ species and the appearance of the spectral feature centred around 989 cm^−1^ corresponding to the Na_2_SO_4_·10H_2_O mirabilite phase. (*d*) Peak heights of the 980 and 989 cm^−1^ fingerprint peaks highlighted in (*c*), showing their respective time dependencies, the time of crystallization *T*_cryst_, the solution saturation temperature *T*_sat_ and the metastable zone width (MSZW): not to be confused with the induction time as this is a polythermal experiment not an isothermal experiment.

The time-dependent diffraction data are presented in Figs. 7 ▸(*a*) and 7 ▸(*b*) for a selected portion of the cooling profile. Here *t*_0_ is the start of the cooling profile at 40°C and data are collected during the −0.2°C min^−1^ cooling ramp. Initially, there were no diffraction peaks due to the absence of solids in the solution prior to crystallization, as the solution was above the saturation temperature. At this time, only the diffuse peak from the aqueous phase was present as a broad band centred on ∼28° 2θ. Subsequently, crystallization was shown by the appearance of sharp 021 and 131 diffraction peaks associated with the metastable mirabilite phase (Na_2_SO_4_·10H_2_O) of sodium sulfate. These data reveal the fast precipitation of the mirabilite phase from the rapid appearance of diffraction peaks in a single 18 s frame collected at ∼1500 s of experiment time, during the cooling step (further plots of these experimental data are provided in Section S5). This indicates that the nucleation mechanism is likely to be instantaneous; all nuclei are formed at the same time point and growth to macroscopic crystals follows (Kashchiev *et al.*, 2010 ▸).

The complementary Raman data are also plotted as a function of time in Fig. 7 ▸(*c*), where the Raman bands associated with the aqueous sulfate ions and mirabilite are found at 980 and 989 cm^−1^, respectively. These data correlate well with the diffraction data; there was an almost immediate appearance of the 989 cm^−1^ Raman band associated with mirabilite, which then rapidly increased in intensity over 20 s. The peak corresponding to the sulfate ions plunged the moment the mirabilite formed and then decreased more gradually as the crystals grew. Following baseline correction, a Gaussian function was fitted to the two peaks, and their respective calculated peak heights and areas were plotted against experimental time [Fig. 7 ▸(*d*)]. These data highlight the fast kinetics of the solution de-supersaturation and the growth of the new crystalline phase. Hence these data allow extraction of the metastable zone width for this from identification of the dissolution and crystallization temperatures/time. More importantly, this experiment highlights the possibility of using the combined XRD–Raman system to quantitatively analyse the concentration of solid and solution species during a crystallization process, giving insight into the pathways and mechanisms by which materials are dissolving, transforming and growing.

## 4. Conclusions

This work has presented the construction and commissioning of a new facility, Flow-Xl, that combines Raman and X-ray diffraction for the laboratory-based *in situ* characterization of crystalline materials in flow systems. Coupling analytical techniques with a range of flow-based sample environments facilitates reproducible measurements and gives access to conditions representative of real-world/industrial settings. The instrument operates with a high-powered rotating Cu (1.54 Å) anode X-ray source, a fast-counting HPC area detector and a dual-laser Raman probe, and allows diffraction and spectroscopic data to be recorded from crystallization systems simultaneously and on timescales of the order of seconds. The facility offers a range of versatile sample environments including batch crystallizers, continuous phase crystallization devices, segmented and droplet phase crystallization devices, and solid-state sample stages, and samples can be translated in *x*, *y* and *z* coordinates relative to the detector position.

Commissioning experiments were conducted for representative inorganic and organic materials, and good-quality reproducible *in situ* quantitative phase information was obtained at relatively short timescales (seconds) for both solid-state and solution-state species using the diffractometer and Raman spectrometer. A representative 0.5 L batch crystallization of sodium sulfate demonstrated that parallel XRD and Raman analyses facilitate rapid phase identification and monitoring of the de-supersaturation of the solution. Both the structural pathway associated with crystallization and the underlying kinetics associated with these processes can therefore be determined. Although synchrotron facilities will always offer higher spatial and temporal resolutions, which can be critical when analysing very rapid crystallization processes, this work has shown that laboratory-based facilities can yield valuable *in situ* data from continuous flow in a matter of seconds, providing an alternative to SR experiments.

## Supplementary Material

Supporting information. DOI: 10.1107/S1600576724006113/iu5051sup1.pdf

## Figures and Tables

**Table 1:** The *d*-spacing refinement of peak positions from Cu *K*α_1,2_ diffraction of a silver behenate sample, highlighting the fitted experimental peak positions and the calculated peak positions from the known crystal structure

Peak (*hkl*)	Fitted position (°, 2θ)	Calculated position (°, 2θ)	Δ position (°, 2θ)
002	3.0352	3.0266	0.0086
003	4.536	4.5406	0.0046
004	6.0376	6.0554	0.0178
005	7.5402	7.5712	0.0310
006	9.044	9.0883	0.0443
007	10.5495	10.6071	0.0576
Mean	–	–	0.0273

**Table 2:** Statistical error analysis showing the RSD of Raman data for the calibration standards of KNO_3_ aqueous solutions and β-L-glutamic acid form slurries

Concentration	Standard deviation/peak area	RSD/peak area %	Standard deviation/peak height	RSD/peak height %
KNO_3_ solutions				
0.625 g L^−1^	32.54	5.55	2.53	3.24
1.25 g L^−1^	29.80	2.81	3.50	2.45
2.50 g L^−1^	213.55	5.85	25.33	5.25
100 g L^−1^	278.14	0.55	48.81	0.74
Mean	–	3.69	–	2.92

L-Glutamic acid slurries				
0.5 wt%	278.83	5.28	25.90	4.48
1.0 wt%	365.87	3.42	9.54	0.77
4.0 wt%	415.40	1.20	39.76	0.86
Mean	–	3.30	–	2.03
