# Supplementary material for: Flow-Xl: a new facility for the analysis of crystallization in flow systems
Source: J Appl Crystallogr. 2024 Aug 19;57(Pt 5):1299–310. doi: 10.1107/S1600576724006113 (PMC11460381; doi:10.1107/S1600576724006113)
Supplement: Supplementary file 1 [file j-57-01299-sup1.pdf]

JOURNAL OF
APPLIED
CRYSTALLOGRAPHY

Volume 57 (2024)

Supporting information for article:

Flow-XI: a new facility for the analysis of crystallization in flow systems

T. D. Turner, C. O'Shaughnessy, X. He, M. A. Levenstein, L. Hunter, J. Wojciechowski, H. Bristowe, R. Stone, C. C. Wilson, A. Florence, K. Robertson, N. Kapur and F. C. Meldrum

This supporting information provides further details of the work highlighted in the main manuscript and is divided into the following sections;

- S1 Humidity cell drawings
- S2 Additional calibration data for the X-ray System
- S3 Additional calibration data for the Raman system
- S4 Details of the error analysis applied to the Raman data
- S5 Additional *in situ* diffraction and Raman data collected during cooling crystallisation of sodium sulphate in a stirred batch crystalliser

S1. Humidity Cell Technical Drawings

Computer aided design drawings of the assembled variable humidity cell; highlighting the kapton/glass window system; the sample chamber to hold powder samples; a cross section showing the channel used to link the humidity feed to the central sample chamber.

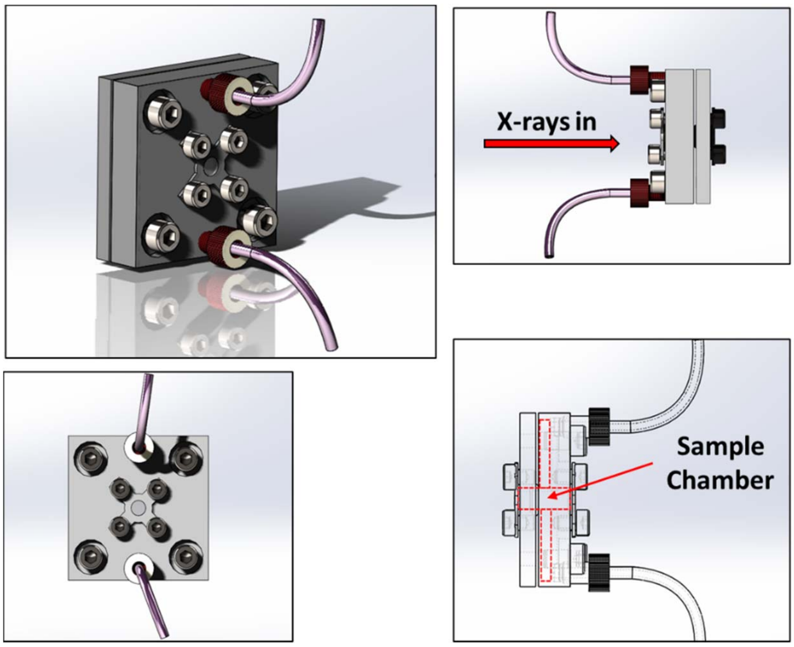

Figure S1 Computer aided design drawings of a variable humidity sample chamber for in situ diffraction and Raman experiments

S2. Additional Slurry XRD Calibration Data for Theophylline Form II

Supplementary diffraction data for low slurry concentrations of the drug molecule theophylline form II in isopropanol suspensions; highlighting the low detection limit of the diffraction instrument for *in-line* analysis.

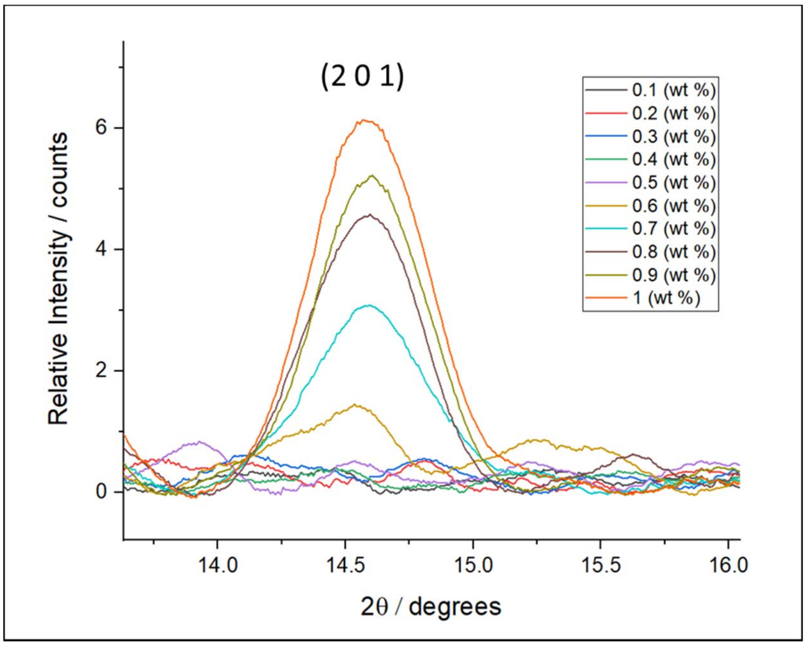

Figure S2 XRD calibration data for theophylline form II at low slurry concentration ranges; 0.1- 1.0 wt%, highlighting the concentration dependence of the (2 0 1) peak in the diffraction pattern; indicating the limit of detection to be 0.6 wt%

S3. Further Raman Calibration Data for Slurry and Solution Species

Supplementary Raman calibration data for calcite aqueous slurries showing good linear correlation between slurry wt% and both peak height and integrated peak area; the data also displays the lower limit of possible detection for calcite under the experimental conditions measured. Additionally, the repeatability of the measurements is provided through statistical analysis of fitted peak heights and integrated peak areas over five repetitions of the same concentration; found to be 2.3 and 3.08 respectively.

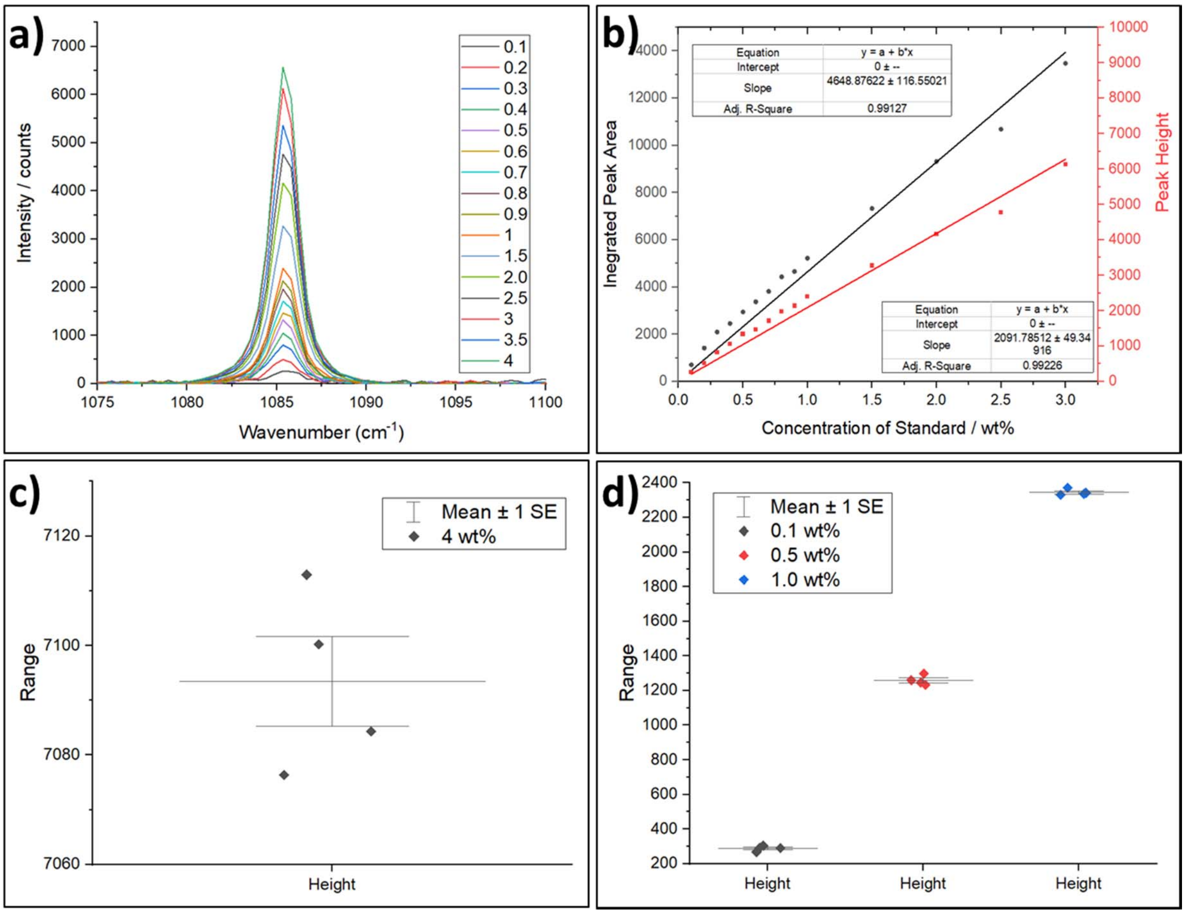

Figure S3 Raman calibration data for calcite aqueous slurries; a) raw Raman data as a function of slurry concentration (wt%), b) linear calibration curves of integrated peak area and peak heights of the 1085 cm^{-1} in a) of the calcite peak vs concentration of standard, c) + d) relative standard deviation of peak heights for five repetitions across three concentrations to highlight the repeatability of the Raman data.

Supplementary Raman calibration data for paracetamol – ethanol solutions and sodium sulphate aqueous solutions showing peak height as a function of concentration. The linear correlation for both systems were found to be good; with R2 values of 0.99 for all fits.

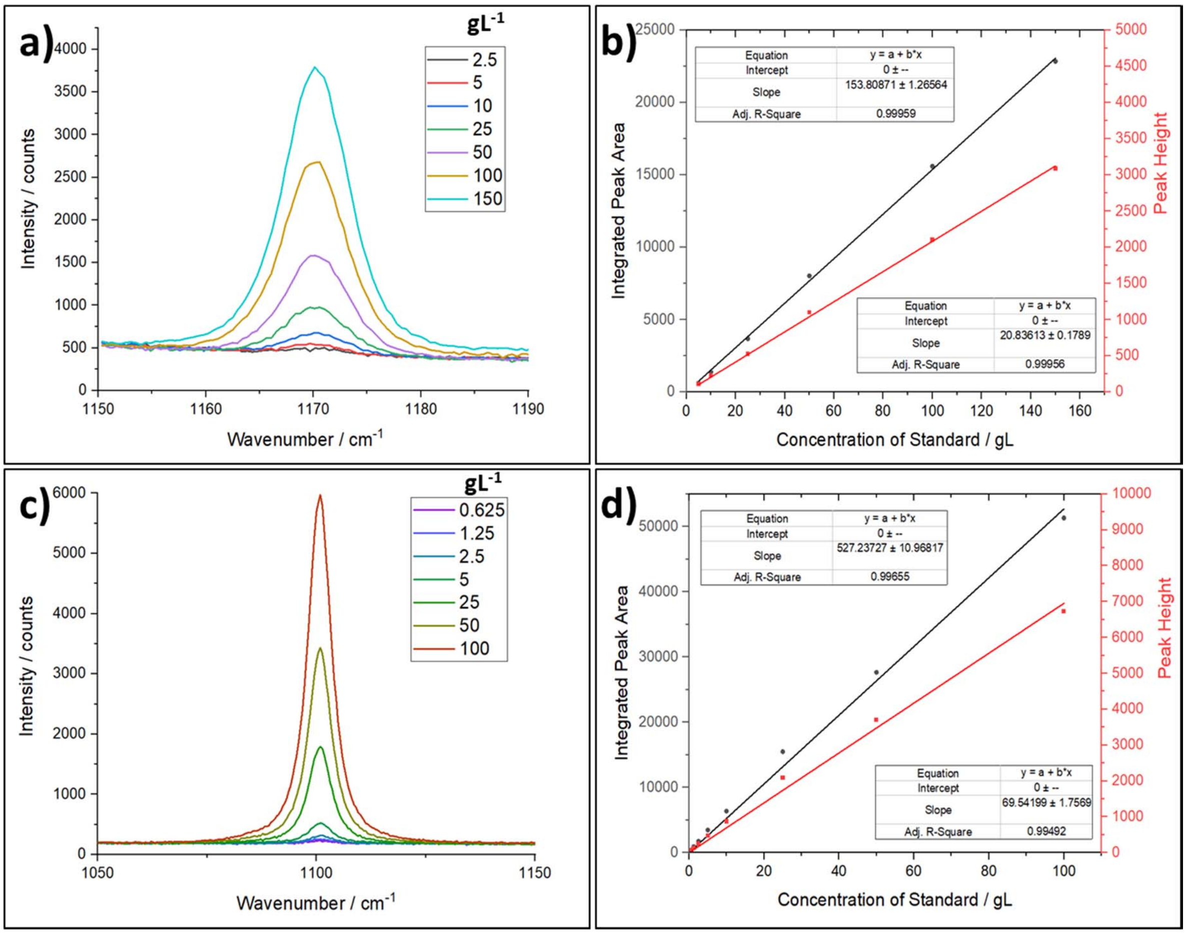

Figure S4 Raman calibration data for solution systems a) paracetamol form 1 dissolved in dry ethanol (gL^{-1}) raw data as a function of concentration centred on the peak at 1170 cm^{-1} b) linear calibration curves of integrated peak area and peak heights as a function of solution concentration for paracetamol ethanol solution data using the data from the 1170 cm^{-1} peak c) Na_2SO_4 aqueous solutions raw Raman data as a function of concentration (gL^{-1}) d) linear calibration curves for the Na_2SO_4 aqueous solution data using the integrated 1100 cm^{-1} peak

S4. Error Analysis using Descriptive Statistics for Raman Calibration Data

This section highlights how the Raman and diffraction data were treated following peak integration using descriptive statistics to highlight the reproducibility of the two techniques in their current set-ups for measuring the concentrations of solid and solution species in flowing crystallisation systems.

Table S1 Sensitivity analysis for the Raman system highlighting the limit of detection (for the systems and concentrations analysed) and relative standard deviation (RSD) of integrated peak heights and widths

Raman	System	Concentration	LOD	RSD Area %	RSD Height %
**Solutions**	KNO_3	0.625 - 100.0 g/L	0.625	3.69	2.92
	Anhydrous Na_2SO_4	0.625 - 100.0 g/L	0.625		
	Paracetamol Form I	2.5 - 5.0 g/L	2.5		
**Slurrys**	Calcium Carbonate Calcite (particle size >50μm)	0.01 - 4.0 wt%	0.02 - 0.06	3.08	2.3
	L-glutamic acid β form (particle size >100μm)	0.1 - 6.0 wt%	0.1	3.3	2.03

$$\sigma = \sqrt{\frac{\sum_{i=1}^{n}(x_i - \mu)^2}{n}}$$

Equation S1 standard deviation (σ) is calculated as the square root of the sum of squared differences of each data point (xi) from the population mean (μ) and divided by the size of the population of data points (n).

$$RSD = \left[\frac{100 \times \sigma}{\mu}\right]\%$$

Equation S2 relative standard deviation (RSD) is calculated as a percentage value of the division of the standard deviation (σ) over the population mean (μ).

S5. Additional Raman and Diffraction Data for the Cooling Crystallisation of Sodium Sulphate

Supplementary plots of the time/temperature dependant Raman and diffraction data collected during the cooling crystallisation of sodium sulphate in a stirred batch crystalliser. The data highlights the sensitivity of the two techniques to detect the nucleation time and onset of crystal growth.

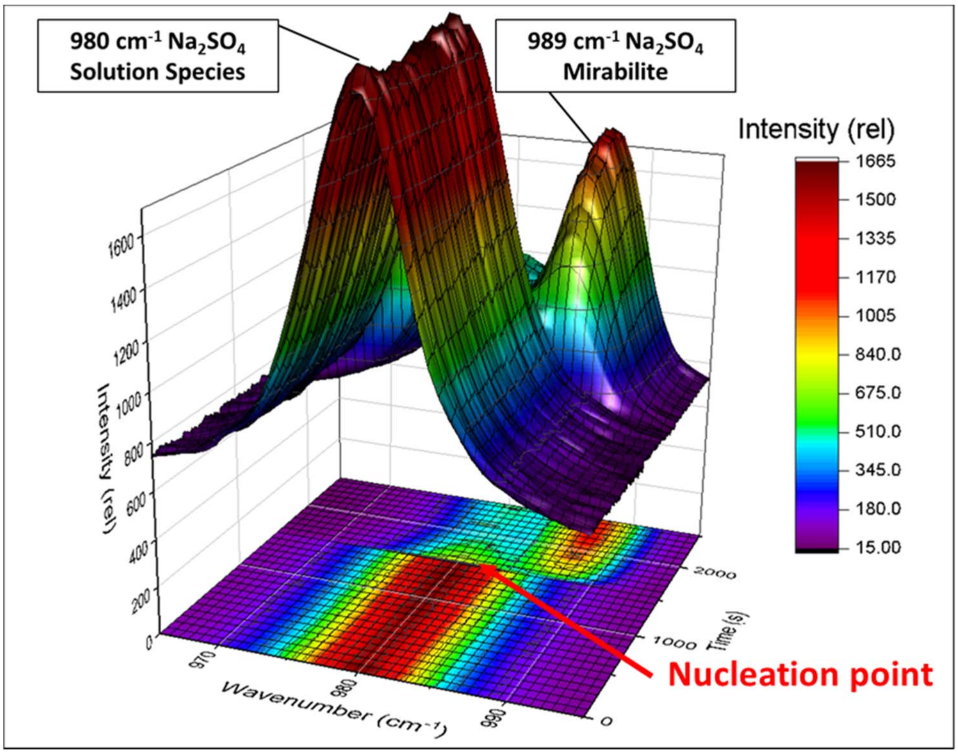

Figure S5 Raman data collected during the cooling crystallisation cycle of sodium sulphate from aqueous solutions; highlighting the solution state species of sodium sulphate, the nucleation point, followed by growth of the metastable mirabilite phase of sodium sulphate.

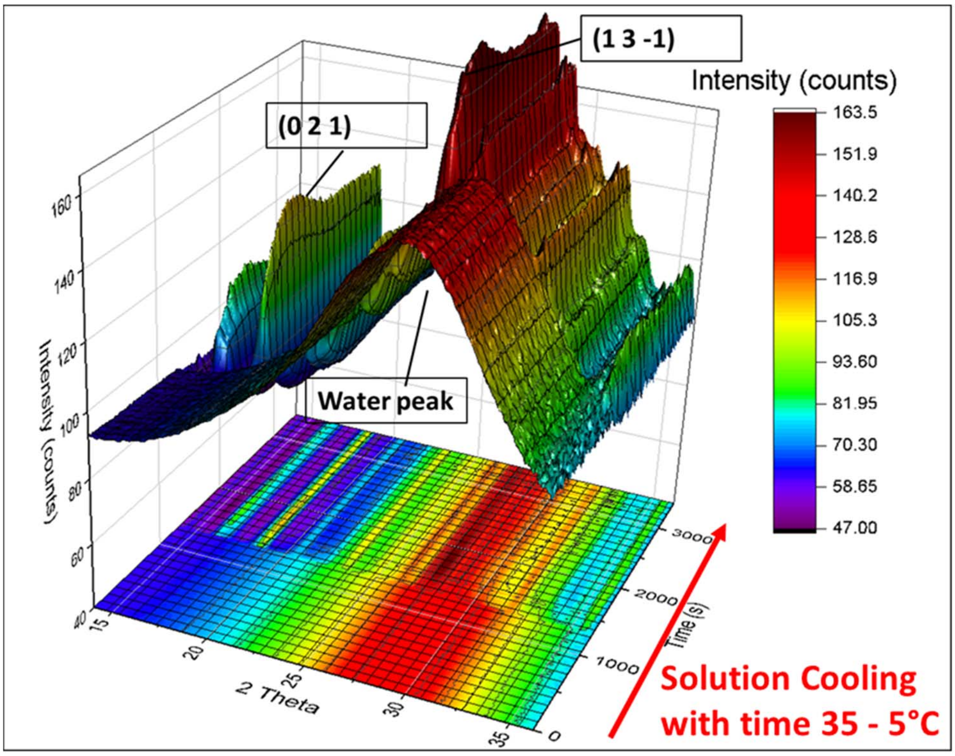

Figure S6 Diffraction data collected during the cooling crystallisation cycle of sodium sulphate from aqueous solution, indicating the water peak prior to nucleation and the subsequent instantaneous appearance of the solid-state diffraction peaks relating to the metastable mirabilite phase of sodium sulphate.
